# Postoperative Organ Dysfunction Risk Stratification Using Extracellular Vesicle-Derived circRNAs in Pediatric Congenital Heart Surgery

**DOI:** 10.3390/cells13171417

**Published:** 2024-08-25

**Authors:** Fahd Alhamdan, Koichi Yuki

**Affiliations:** 1 Department of Anesthesiology, Critical Care and Pain Medicine, Cardiac Anesthesia Division, Boston Children’s Hospital, Boston, MA 02115, USA; 2 Departments of Immunology and Anaesthesia, Harvard Medical School, Boston, MA 02115, USA; 3 Broad Institute of MIT and Harvard, Cambridge, MA 02142, USA

**Keywords:** Evs, circRNA, Pediatrics, congenital heart surgery, organ dysfunction

## Abstract

Breakthroughs in surgical and medical techniques have significantly improved outcomes for children with congenital heart disease (CHD), but research continues to address the ongoing challenge of organ dysfunction after surgery, particularly in neonates and infants. Our study explored circular RNAs (circRNAs) within plasma-derived extracellular vesicles (EVs) in neonates and infants undergoing CHD surgery. Post-surgery EV circRNAs showed dramatic expression changes between organ dysfunction (OD) and control groups. Tissue injury-related pathways were consistent across pre- and post-surgery in OD. The top two significant predicted tissue sources of these circRNAs originated from the respiratory system, aligning with the fact that all patients in the OD arm experienced respiratory dysfunction. Five of these circRNAs, namely circ-CELSR1, circ-PLXNA1, circ-OBSL1, circ-DAB2IP, and circ-KANK1, significantly correlated with PELOD (Pediatric Logistic Organ Dysfunction) score and demonstrated high performance (AUC = 0.95), supporting the potential of circRNAs as prognostic markers. These findings pave the way for EV circRNAs as promising tools for managing post-surgical organ dysfunction and potentially guiding therapeutic strategies in children with CHD.

## 1. Introduction

Despite significant improvements in overall outcomes for patients with congenital heart diseases (CHDs), neonates and infants undergoing cardiac surgery still experience high rates of postoperative complications and mortality. The Kids’ Inpatient Database (KID) reports an in-hospital mortality rate of 6.9% for neonates and infants, compared to 1.28%, 0.67%, and 0.83% for the age groups 1–5, 6–12, and 13–17 years old, respectively, [1]. Longer cardiopulmonary bypass (CPB) time [1] and larger blood transfusion requirements [2,3,4] are established clinical risk factors. These factors contribute to the development of thrombotic complications and subsequent organ dysfunction/failure, which are major contributors to worse outcomes [5]. Understanding the mechanisms underlying these complications is therefore critical.

Extracellular vesicles (EVs) are membrane-enclosed nanoparticles that act as messengers between cells. These spheres encapsulate a diverse cargo of functional proteins, nucleic acids, lipids, and other biochemical molecules, enabling them to influence various cellular processes [6]. Their pleiotropic role has been implicated in a range of diseases [7,8,9].

Among different RNA species encapsulated within EVs, circular RNAs (circRNAs) represent a recently discovered class of non-coding RNAs with exciting potential for disease diagnosis [10]. Their unique closed-loop structure confers exceptional stability, making them resistant to degradation and facilitating their detection in biological samples [11]. Additionally, the tissue-specific expression patterns of circRNAs offer a remarkable advantage for pinpointing the origin of pathological processes within the body [12]. CircRNAs harbor a variety of functions including microRNA sponging, cell cycle regulation, and cellular communications [13]. Thus, in this study, we examined the expression profiles of plasma EV circRNAs in neonates and infants who developed organ dysfunction (respiratory failure) following congenital cardiac surgery and explored different associations of circRNAs with the pathomechanism of this condition.

## 2. Material and Methods

### 2.1. Study Design and Setting

This single-center prospective cohort study was conducted at a quaternary academic pediatric medical center. The study was approved by the Institutional Review Board at Boston Children’s Hospital, and written informed consent was obtained from a parent or a legal guardian. We followed the Strengthening the Reporting of Observational Studies in Epidemiology (STROBE) statement for cohort studies in the preparation of this manuscript [14].

### 2.2. Patient Selection and Perioperative Course

We included neonates and infants scheduled for congenital cardiac surgery on CPB. Exclusion criteria included patients who did not necessitate CPB, were presented with an active infection, received chronic steroid therapy, had an immunodeficiency such as human immunodeficiency virus (HIV) infection, or had a history of malignancy. The patients were enrolled from 31 May 2022 to 22 February 2023.

All patients in the study underwent general anesthesia with endotracheal intubation and arterial and central venous line placement. General anesthesia was provided by the combination of volatile anesthetics, opioids, and benzodiazepines at the discretion of staff anesthesiologists. After surgical dissection, patients were heparinized and underwent aortic and venous cannulation for CPB. The CPB circuit was primed with one unit of packed red blood cells (pRBC) and one unit of fresh frozen plasma (FFP), maintaining a target hematocrit level greater than 30% per our institutional standard protocol. The use of circulatory arrest or regional perfusion, temperature management, and modified ultrafiltration (MUF) was determined on a case-by-case basis. In cases of non-surgical microvascular bleeding, platelets and cryoprecipitate were administered. Neonates and infants typically did not receive FFP transfusion after CPB in our institution. Following surgery, patients were kept intubated and transferred to the intensive care unit (ICU) for postoperative care.

### 2.3. Clinical Data Collection

Demographic information, comorbidities, diagnosis, procedures, laboratory values, CPB details, type and volume of blood products administered, postoperative complications, duration and type of respiratory support, vital signs, as well as hospital and ICU lengths of stay were extracted from the electronic medical record for the analysis of clinical data. The presence of organ dysfunction/failure and/or thrombosis was collected as postoperative complications. Due to the absence of a standardized definition for organ dysfunction following congenital cardiac surgery, we adopted previously published criteria established by others [15,16]. Organ dysfunctions included the following: (1) cardiovascular dysfunction (low cardiac output syndrome, reliance on a vasoactive drug to maintain blood pressure, or two of the following: metabolic acidosis, elevated arterial lactic acid, oliguria, or prolonged capillary refill); (2) respiratory dysfunction (arterial oxygen tension/fraction of inspired oxygen (PaO_2_/FiO_2_) < 300, arterial carbon dioxide tension (PaCO_2_) > 65 torr or 20 mmHg over baseline PaCO_2_, need for >50% FiO_2_ to maintain oxygen saturation ≥ 92%, or need for non-elective mechanical ventilation, prolonged mechanical ventilation ≥ 5 days; (3) renal dysfunction (the presence of creatinine of >114 μmol/L, urine output of <1 mL/kg/h despite diuretic administration, or the requirement for ultrafiltration or hemodialysis; (4) coagulopathy or bleeding complication requiring chest exploration for bleeding or removal of clots, intracranial hemorrhage, prothrombin or partial thromboplastin time was three times normal, or >30 mL/kg of blood products were infused during a 24 h period; (5) central nervous system dysfunction (development of a new intracranial infarct or hemorrhage, evidence of hypoxic-ischemic injury by clinical examination or computed tomography of the head, or brain death); and (6) hepatic dysfunction (bilirubin concentration of >2 mg/dL and/or increase in hepatic cellular enzymes two or more times normal). Thrombosis was defined as the presence of any vascular thrombosis detected using ultrasound diagnostic imaging. In addition, we also calculated Pediatric Logistic Organ Dysfunction-2 (PELOD-2) score to assess organ dysfunction as previously used in the CHD cohort [17], although recognizing its limitation because this system cannot differentiate between therapy and severity of diseases from cardiovascular standpoint [18]. Organ dysfunction type, surgical procedure type, and other demographic parameters for each study subject were listed in Supplementary Table S1.

### 2.4. Blood Sample Collection

Blood samples were collected from patients through existing central venous catheters. The blood was obtained at the following time points: (1) immediately after anesthesia induction (pre-surgery), (2) on postoperative day 1 (post-surgery). Once blood samples were received, they were subjected to centrifugation at 1000× *g* for 10 min to obtain plasma. Plasma was immediately stored at −80 °C.

### 2.5. Plasma Collection and EVs Isolation

EVs were isolated from 200 to 300 µL of the plasma samples using the exoEasy Midi kit (Qiagen, Hilden, Germany) according to the manufacturer’s instructions. In brief, plasma samples were thawed at 37 °C and centrifuged at 10,000× *g* for 12 min at room temperature to remove cellular debris. Plasma samples were mixed with XBP buffer and added to the exoEasy spin column. Columns were washed with XWP buffer, and then EVs were eluted from the column membrane using XE buffer.

### 2.6. Nanoparticle Tracking Analysis (NTA)

NTA was performed using the ZetaVIEW equipment (Particle Metrix, Ammersee, Germany). Initially, the instrument was rinsed using 5 mL of filtered water to remove any remaining particles. Auto alignment was conducted by injecting 2 mL of polystyrene particles into the instrument, which was rinsed again afterwards. As optimal particle per frame values ranges from 140 to 200 particles/frame, plasma EV samples were diluted 1:500 in pre-filtered PBS. Subsequently, 1 mL of each diluted EV sample was injected into the instrument which was carefully rinsed after each measurement.

The manufacturer’s default software settings for EVs, liposomes or nanospheres were selected accordingly. For each measurement, two video cycles were recorded by scanning 11 cell positions and capturing 30 frames per position with the following settings: Focus: autofocus; Camera sensitivity for all samples: 75; Shutter: 70; Scattering intensity: detected automatically; Cell temperature: 25 °C; pH: 7.0. After capture, the videos were analyzed by the in-built ZetaView Software v.8.05.05 SP2 with specific analysis parameters: Max size: 1000; Min size: 5; Min brightness: 25. Hardware: embedded laser: 40 mW at 488 nm; camera: CMOS.

### 2.7. EV Proteomic Antibody Array

EV markers including Golgi matrix protein 130 (GM130), CD63, CD81, epithelial cellular adhesion molecule (EpCAM), annexin A5 (ANXA5), tumor susceptibility gene 101 (TSG101), flotillin 1 (FLOT1), intercellular adhesion molecule 1 (ICAM1), and ALG-2 interacting protein X (ALIX) were measured using a Western blot-based method: Exo-Check Exosome Antibody Array Kit, Human (System Biosciences, Palo Alto, CA, USA). In brief, according to the manufacturer’s instruction, 50 µg of eluted EVs were lysed, labeled, and incubated overnight with a panel-antibody-coated membrane. On the next day, the membrane was incubated with a detection antibody and developed with a 1:1 substrate mixture KwikQuant Western Blot Detection kit (Kindle Biosciences, Greenwich, CT, USA). Subsequently, the membrane was visualized with KwikQuant Pro Imager (Kindle Biosciences).

### 2.8. EV RNA Extraction

Total RNA including small RNAs was extracted from EVs using miRNeasy kit (Qiagen, Hilden, Germany) according to the manufacturer’s instructions. RNA yield was measured by Qubit™ microRNA Assay Kit (Thermo Fisher Scientific, Waltham, MA, USA). RNA size distribution and quality were assessed by Bioanalyzer Small RNA Analysis kit (Agilent Technologies, Santa Clara, CA, USA).

### 2.9. RNA Library Preparation and Small RNA Sequencing

Small RNA libraries were constructed using NEBNext Small RNA Library Prep Set for Illumina (New England Biolabs, Ipswich, MA, USA), according to the manufacturer’s protocol, with minor modifications for the low RNA input. Briefly, 3 ng of RNA was used for the library preparation. The 3′ SR Adapter, SR RT Primer, and 5′ SR Adapter were diluted 1:4, and the RNA was ligated with both adapters, and was reverse transcribed, barcoded, and amplified for 15 cycles. The generated libraries were cleaned up using AMPure XP Beads (Beckman Coulter, Brea, CA, USA) and quantified using the Qubit™ dsDNA HS Assay (Thermo Fisher Scientific) and the Bioanalyzer High Sensitivity DNA Analysis kit (Agilent Technologies) prior to sequencing on a HiSeq4000 platform (Illumina, San Diego, CA, USA) with High Output Kit v2.5 and 50 bases single-reads, according to the manufacturer’s instructions.

### 2.10. Bioinformatic Analysis

CircRNA sequences and annotations were retrieved from the circBase 0.1 database [19] for the human genome assembly hg19. Small RNA-Seq reads were processed using miRMaster 2.0 [20]. This included quality control checks to assess read quality and the removal of Illumina adapter sequences. Subsequently, meticulously clean reads were mapped to the reference genome (hg19 assembly) and counted to quantify transcript abundance. To filter out low abundant circRNAs, expression cut-off was applied >5 counts for at least 4 samples. Differential expression analysis was conducted with Deseq2 R package (version 1.38.3). Density analyses were computed using the average expression of the top 500 variable circRNAs per each sample and visualized with ggdensity R package (version 1.0.0). Functional analysis, including biological pathways and processes, was curated with the implementation of different databases such as Reactome [21,22], Biological process (Gene Ontology) [22], NCATS BioPlanet [23] by utilizing clusterProfiler package (Version 4.6.2). Cell type and tissue specificity were retrieved from HuBMAP ASCTplusB augmented 2022 database [14]. Corrplot R package (version 0.92) was utilized to create correlation analysis and visualization. ROC-AUC analysis was computed with pROC R package (version 1.18.5).

### 2.11. Statistical Analysis

Data were shown as mean ± S.D. Student’s unpaired *t*-test were used for statistical significance with *p* value * < 0.05, ** < 0.005, and *** < 0.001.

## 3. Results

### 3.1. Altered Plasma EV circRNA Expression Profiles in Individuals with Respiratory Dysfunction Following Pediatric Congenital Heart Surgery

Our current work included four neonates and infants who developed organ dysfunction (OD), primarily respiratory dysfunction, following congenital cardiac surgery, and as controls we enrolled five participants who did not develop organ dysfunction (NOD). The characteristics of all neonates and infants enrolled for this study are shown in Table 1.

To probe the complex pathomechanisms associated with postoperative OD, we employed EV circRNAs due to their versatile biological functions and their intriguing tissue specificity.

**Figure 1:**
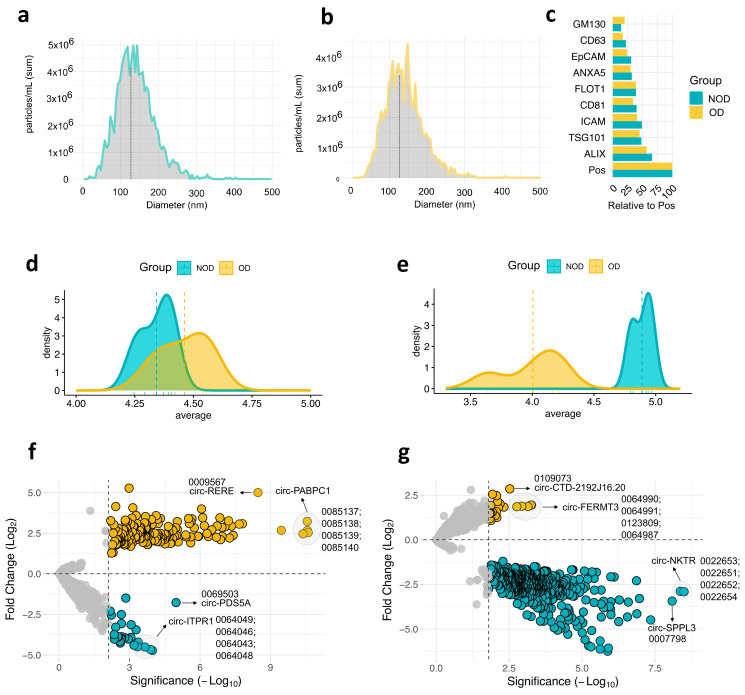
Expression profiles of plasma EV circRNAs during pediatric congenital cardiac surgery. Nanoparticle Tracking Analysis (NTA) of size distribution of EVs isolated from (**a**), organ dysfunction (OD) samples (*n* = 4) and (**b**), non-organ dysfunction (NOD) samples (*n* = 5). (**c**), EV proteomic antibody array measuring the levels of eight EV protein markers. Pos: Positive control, GM130: cellular contamination marker. Density plots depict the average expression of the top 500 variable circRNAs of OD and NOD at (**d**), pre-surgical time point and (**e**), post-surgical time point. Volcano plots of differential expressed circRNAs in OD compared to NOD at (**f**), pre-surgical time point and (**g**), post-surgical time point.

Next, we isolated EVs from the plasma of all individuals at two time points: pre-surgery, after anesthesia induction (baseline); and post-surgery, on postoperative day 1. Subsequently, we characterized the EVs size and protein markers. Nanoparticle analysis (NTA) revealed a median EV size less than 150 nm, which falls within the typical range for exosomes [15] (Figure 1a,b). This was further observed by examining a panel of EVs of established EVs protein markers (Figure 1c and Supplementary Figure S1). All markers were detected in both study groups, with ALIX and TSG101 (exosomes markers) showing the highest expression.

We then conducted small RNA-Sequencing (RNA-Seq) analysis to comprehensively examine the circRNA cargo within these vesicles. Analysis of average circRNA expression revealed a remarkable overlap between the OD and NOD groups in the pre-surgery samples (Figure 1c). However, a dramatic shift in the post-surgery OD group was observed, leading to a clear distinction from the post-surgery NOD group (Figure 1d). This indicates a potential association between the surgical procedure and a negative outcome, including the possibility of postoperative OD.

To address the differences between OD and NOD at the two time points of the congenital cardiac surgery, we computed the differentially expressed (DE) circRNAs for the two pairwise comparisons (Figure 1f, g and Supplementary Table S2). By setting the threshold for false discovery rate (FDR) < 0.05, OD versus NOD revealed 229 upregulated and 36 downregulated circRNAs in pre-surgery (Figure 1f), and 39 upregulated and 643 downregulated circRNAs in post-surgery (Figure 1g), mirroring the previously observed post-surgical decline in circRNA expression profiles within the OD group (Figure 1d). As shown in the volcano plots, it is noteworthy that a single gene can serve as a template for the generation of several circRNAs.

To account for the larger transfusion volumes in the OD group (Table 1), we evaluated the housekeeping microRNAs (miRNAs) expression levels from the sequencing data [16,17] (Supplementary Table S3). No significant changes in miRNA expression were observed between the OD and NOD groups, suggesting that the transfusion volume did not introduce a dilution bias.

### 3.2. Biological Pathways and Processes Associated with the Altered EV circRNAs

**Figure 2:**
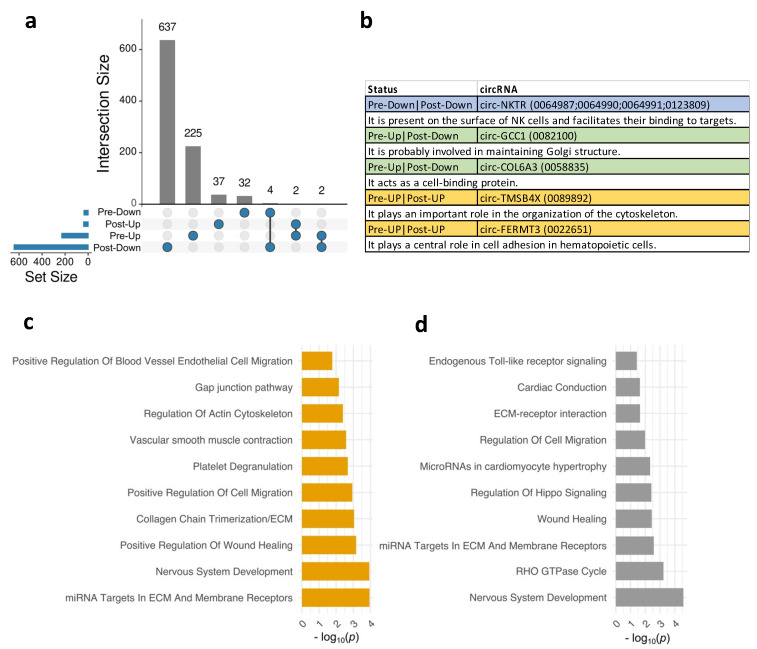
Biological pathways and functions enriched for plasma EV circRNAs in OD versus NOD at pre- and post-surgical time points during pediatric congenital cardiac surgery. (**a**) Upset diagram showing unique and shared EV circRNAs in OD versus NOD across the two time points. (**b**) Table of the shared EV circRNAs in (**a**) with the corresponding functions of their associated genes. Bar plots exhibiting the top 10 significant biological pathways (*p* < 0.05) for the EV circRNAs-associated genes in OD versus NOD at (**c**) pre-surgical time point and (**d**) post-surgical time point.

Next, we aimed to explore the shared and unique DE circRNAs in OD compared to NOD at pre- and post-surgical time points. An Upset diagram (Figure 2a) visually depicted the limited overlap of DE circRNAs in OD versus NOD groups at pre- and post-surgery. Interestingly, among the few shared downregulated DE circRNAs, there were four originating from the NKTR gene (Natural Killer Cell Triggering Receptor). This gene encodes a surface receptor on natural killer (NK) cells, crucial for their binding to target cells [18] (Figure 2b). Other shared circRNAs included circ-TMSB4X (Thymosin Beta 4 X-Linked) and circ-FERMT3 (FERM Domain Containing Kindlin 3) upregulated at both time points (Figure 2a,b). The upregulated circRNAs in pre-surgery that were downregulated in post-surgery were circ-GCC1 (GRIP And Coiled-Coil Domain Containing 1) and circ-COL6A3 (Collagen Type VI Alpha 3 Chain).

Then, we assessed the biological pathways and processes associated with the host genes of the DE circRNAs in OD versus NOD at pre- and post-surgery (Figure 2c,d) (Supplementary Table S4). Enriched pre-surgery pathways primarily revolved around vascular endothelial cell remodeling and migration (Figure 2c), including gap junction pathway, positive regulation of blood vessel endothelial cell migration, and regulation of actin cytoskeleton. This might indicate that vascular leak or injury was present prior to surgical intervention. While some pathways related to vascular function remained enriched in the post-surgery DE circRNAs (Figure 2d), there was a notable emergence of distinct pathways, such as RHO GTPase cycle, endogenous Toll-like receptor signaling, and regulation of hippo signaling. The latter is recognized to impact cell proliferation and tissue repair after injury [24].

### 3.3. Tissue Specificity of the Altered EV circRNAs and Their Potential Clinical Associations

**Figure 3:**
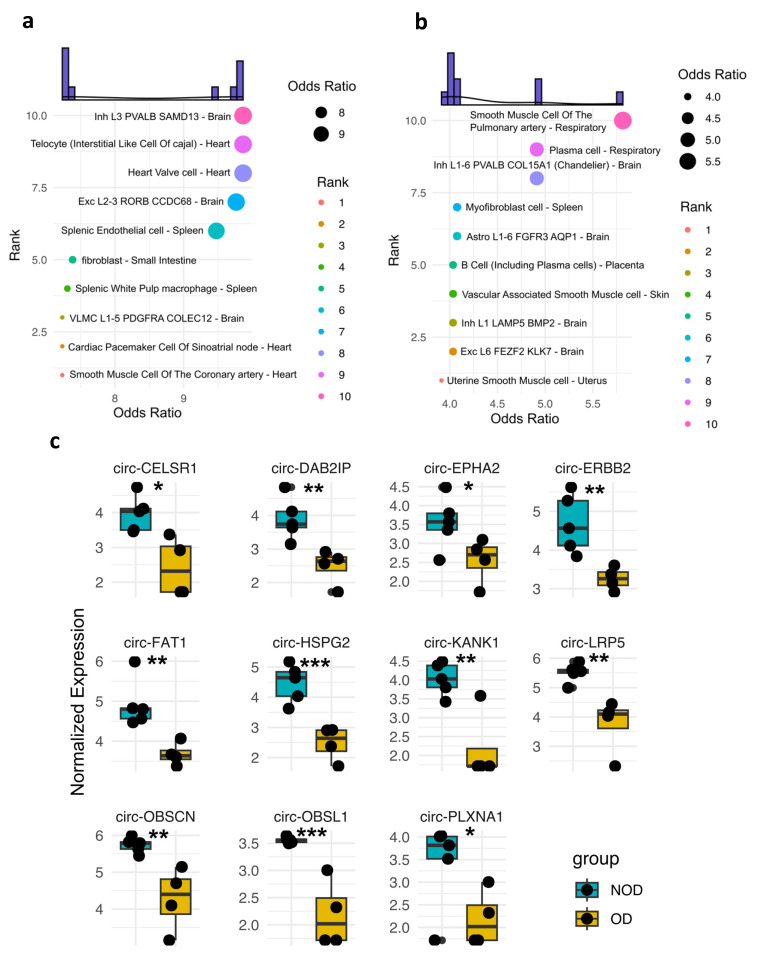
Tissue-specificity of the enriched plasma EV circRNAs in OD versus NOD at pre- and post-surgical time points during pediatric congenital cardiac surgery (**a**–**c**). Dot plots exhibiting the predicted tissue and cellular sources of the EV circRNAs-associated genes in OD versus NOD at (**a**) pre-surgical time point and (**b**) post-surgical time point. (**c**) Box plot of the normalized expression levels of respiratory system-derived circRNAs in OD compared to NOD. Statistical analysis was performed using Student’s *t* test. *, ** and *** denote *p* < 0.05, <0.005, and <0.001, respectively.

A key advantage of circRNAs is their ability to harbor tissue-specific molecular signatures [12], making them potentially valuable for diagnostic purposes. Thus, we sought to examine the potential tissue/cellular sources of DE circRNAs in OD versus NOD at pre- and post-surgery samples (Figure 3a,b). The pre-surgery DE circRNAs were predominantly predicted to originate from specific cell types within the heart and brain (Figure 3a). A striking observation emerged in the post-surgery DE circRNAs analysis (Figure 3b). The top two predicted tissue sources shifted to include the respiratory system, which is particularly noteworthy in the context of the postoperative respiratory dysfunction observed in the OD group. This finding suggests a potential involvement of the respiratory system-derived circRNAs in the post-surgical response, particularly for individuals who develop respiratory dysfunction.

We then overlaid the expression profiles of the DE circRNAs associated with the two predicted cellular sources of the respiratory system (Figure 3c). As anticipated, all those circRNAs displayed significantly lower expression levels in OD compared to NOD, matching the decline in the respiratory function in the OD group.

**Figure 4:**
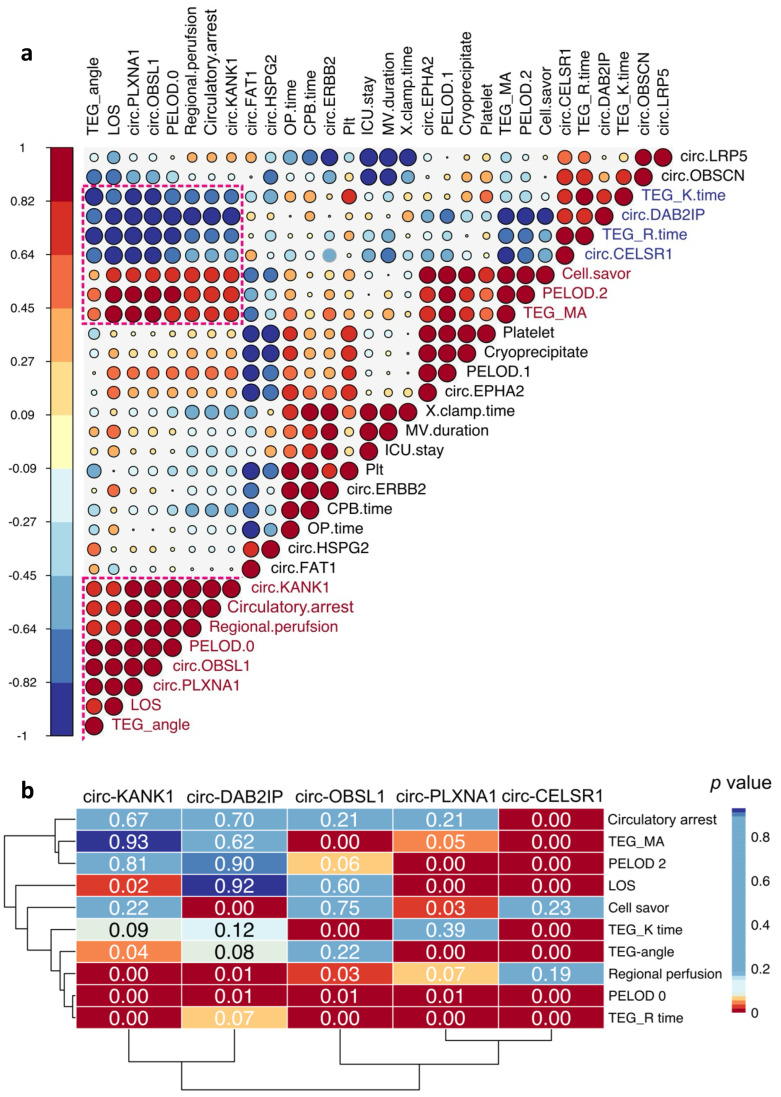
Correlation analysis of the respiratory system-derived circRNAs and surgical/clinical parameters. (**a**) Correlation analysis of the 11 respiratory system-derived circRNAs and several clinical and surgical parameters depicted by the coefficient scores (1, –1). (**b**) Statistical significance of the correlations between circRNAs and clinical/surgical parameters in the three selected clusters from (**a**).

To infer the potential clinical associations of these respiratory system-derived circRNAs, we conducted correlation analysis between these circRNAs and a panel of clinical and surgical parameters (Figure 4a). Notably, the first cluster from the bottom contained the highest number of circRNAs and exhibited robust positive and negative correlations with circRNAs in two other clusters.

We further investigated the correlation significance between the circRNAs and clinical parameters within the highlighted correlation blocks (Figure 4a,b). Interestingly, circ-CELSR1 (Cadherin EGF LAG Seven-Pass G-Type Receptor 1) displayed significant negative correlations with most of the clinical and surgical parameters. This suggests a potential role for circ-CELSR1 in the pathophysiology of postoperative respiratory dysfunction. Additionally, we observed a significant correlation between PELOD 0 (Pediatric Logistic Organ Dysfunction) and five of the respiratory system-associated circRNAs (Figure 4b). The ROC-AUC (Receiver Operating Characteristic–Area Under the Curve) of these five circRNAs exhibited AUC score of 0.95 (Supplementary Figure S2), indicating potential links between these clinical parameters and the overall circRNA expression profile in the respiratory system following surgery.

## 4. Discussion

In our current study involving individuals with OD, primarily respiratory failure, we revealed that post-surgery circRNAs were predominantly expressed in specific cell types within the respiratory system, particularly smooth muscle cells of the pulmonary artery and plasma cells. Identifying these cell type-specific circRNAs holds promise for developing rapid diagnostic tools and targeted therapies to minimize organ injury. Our observations were further supported by a striking shift in the expression profiles of the circRNAs between pre- and post-surgical time points. Given the half-life of circRNAs can be as short as eight hours [25], this dramatic shift could strongly suggest a link between the surgical procedure and the development of OD. However, we cannot rule out the possibility that pre-existing vascular endothelial cell injury, as indicated by the enriched pre-surgery pathways, was exacerbated by the surgical procedure.

CircRNAs have been implicated as diagnostic tools and therapeutic targets in several cardiovascular studies [26,27,28]. Circ-TMSB4X (0003416) (Thymosin Beta 4 X-Linked) was demonstrated to be a potential diagnostic biomarker in children with pulmonary arterial hypertension (PAH) that was caused by CHD [29]. Consistent with our observations, this circRNA was found to be down-expressed in PAH compared to control subjects. We exhibited that circ-SPPL3 (0007798) (Signal Peptide Peptidase Like 3) was among the top five significant circRNAs to be associated with CHD-related OD. In line with this, circ-SPPL3 was shown to be a candidate biomarker for CHD tetralogy of Fallot [30]. Whether this involves the respiratory system in patients with tetralogy of Fallot warrants further investigation. Targeting of circRNAs was illustrated in different studies. Knockdown of circ-Nfix (Nuclear Factor I X) in mice enhanced cell proliferation and angiogenesis and suppressed apoptosis, thereby promoting cardiac regenerative repair post-myocardial infarction [31]. On the contrary, Circ-Ttc3 (Tetratricopeptide Repeat Domain 3) overexpression in cardiomyocytes suppressed apoptosis by sponging the proapoptotic miR-15b, and thus protected against pathological cardiac remodeling post-myocardial infarction [32].

The correlation pattern between respiratory system-derived circRNAs and surgical/clinical parameters may shed light on potential causes of OD/respiratory dysfunction. All five candidate circRNAs significantly correlated with PELOD 0, indicating a possible association with OD. Additionally, TEG measurements and regional perfusion using CBP seemed to be key risk factors influencing surgical outcomes, including respiratory failure.

The host gene of circ-CELSR1 (Cadherin EGF LAG Seven-Pass G-Type Receptor 1) significantly correlated with all the candidate surgical/clinical parameters and was required for fetal lung devolvement. Its absence caused lung morphogenesis defects [33]. Additionally, circ-CELSR1 sponges miR-598 [34]. Given miR-598 attenuates tissue proliferation, the downregulation of circ-CELSR1 could have increased the role of miR-598. Similarly, circ-PLXNA1 can sponge miR-214 [35]. Because the inhibition of miR-214 attenuates lung injury [36], the reduction in circ-PLAXNA1 can aggravate lung injury by harboring miR-214.

The observed downregulation of the regulation of hippo signaling after surgery further supports the functional significance of circRNAs. This signaling pathway is well-recognized for its critical role in tissue regeneration following injury [37]. Yes-associated protein (YAP), the major component of hippo signaling, has been shown to ameliorate the self-renewal of type II alveolar epithelial cells (AECIIs) and their differentiation into type I alveolar epithelial cells (AECIs) to rescue gas exchange following lung injury [38,39,40].

We acknowledge the limited number of individuals that enrolled in this study, the potential effects of patients’ demographic factors, the type of surgical procedures and other clinical practice on circRNAs expression profiles, and the lack of mechanistic experiments. However, this study underscores the potential of EV circRNAs as prognostic and therapeutic tools for organ dysfunction following congenital cardiac surgery.

## Figures and Tables

**Table 1:** Demographic data for the study participants.

	Organ Dysfunction (OD)	Non-Organ Dysfunction (Ctrl)	*p* Value
Age (mo)	6.53 ± 5.47	6.61 ± 5.79	0.39
Male (%)	10	20	0.27
OP time (min)	487.50 ± 84.80	396.00 ± 137.57	0.14
CPB time (min)	264.75 ± 31.57	196.00 ± 79.46	0.08
X-clamp time (min)	152.25 ± 38.30	83.00 ± 70.74	0.06
Circulatory arrest (%)	25% (1/4)	60% (3/5)	0.18
Regional perfusion (%)	25% (1/4)	60% (3/5)	0.18
MV duration (h)	325.58 ± 368.99	72.58 ± 17.60	0.08
ICU stay (h)	442.75 ± 314.65	99.00 ± 13.02	0.02 *
LOS (d)	41.13 ± 24.20	13.02 ± 6.53	0.02 *
PELOD 0	10.00 ± 2.82	7.40 ± 1.40	0.05 *
PELOD 1	4.25 ± 0.96	5.20 ± 1.10	0.11
PELOD 2	5.00 ± 0.82	4.40 ± 2.51	0.33
Salvaged RBCs (mL/kg)	39.59 ± 17.55	20.62 ± 13.57	0.05 *
Platelets (mL/kg)	27.75 ± 17.75	17.24 ± 5.90	0.12
Cryoprecipitate (mL/kg)	10.41 ± 8.80	7.30 ± 7.16	0.29

Data are expressed as mean ± standard deviation. Significant differences between study groups are indicated by * (*p* ≤ 0.05). Abbreviations: OP: operation; X-clamp: aortic cross clamp; CPB: cardiopulmonary bypass; MV: mechanical ventilation; LOS: length of hospital stay; PELOD score: Pediatric Logistic Organ Dysfunction (severity score); PELOD 0: PELOD score at ICU admission after surgery; PELOD 1: PELOD score on postoperative day 1; PELOD 2: PELOD score on postoperative day 2; RBCs, red blood cells.

## Data Availability

Raw sequencing data will be available on reasonable request from the corresponding author.

## Supplementary Materials

The following supporting information can be downloaded at: https://www.mdpi.com/article/10.3390/cells13171417/s1, Figure S1: Characterization of protein markers. Raw membrane visualization of the EV protein markers of both OD and NOD groups. Figure S2: ROC-AUC analysis of the combined classification score of the five candidate circRNAs (circ-CELSR1, circ-PLXNA1, circ-OBSL1, circ-DAB2IP, and circ-KANK1). Table S1: Age, gender, surgery type, and type of organ dysfunction in the study clinical cohort. Table S2: Differentially expressed circRNAs between OD and NOD at pre- and post-surgical time points. Table S3: Differential expression analysis of housekeeping EV miRNAs in OD versus NOD at post-surgery. Table S4: Biological pathways-associated host genes of circRNAs.
