# Supplementary material for: Postoperative Organ Dysfunction Risk Stratification Using Extracellular Vesicle-Derived circRNAs in Pediatric Congenital Heart Surgery
Source: Cells. 2024 Aug 25;13(17):1417. doi: 10.3390/cells13171417 (PMC11394075; doi:10.3390/cells13171417)
Supplement: Supplementary file 1 [file cells-13-01417-s001.zip › Figure S1.pdf]

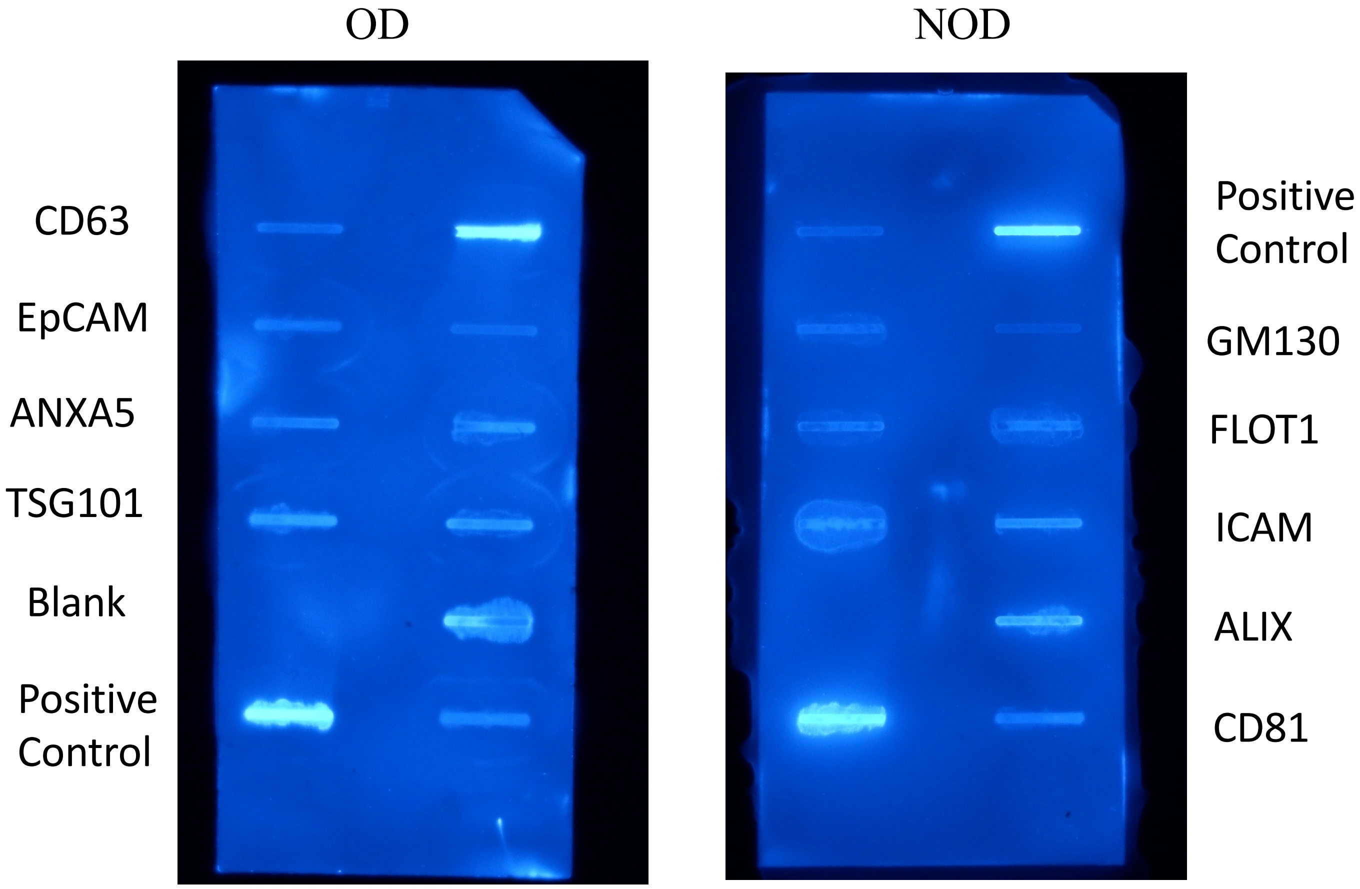

Supplementary Figure S1: Characterization of protein markers. Raw membrane visualization of the EV protein markers of both OD and NOD groups.
