# Supplementary material for: Postoperative Organ Dysfunction Risk Stratification Using Extracellular Vesicle-Derived circRNAs in Pediatric Congenital Heart Surgery
Source: Cells. 2024 Aug 25;13(17):1417. doi: 10.3390/cells13171417 (PMC11394075; doi:10.3390/cells13171417)
Supplement: Supplementary file 1 [file cells-13-01417-s001.zip › Figure S2.pdf]

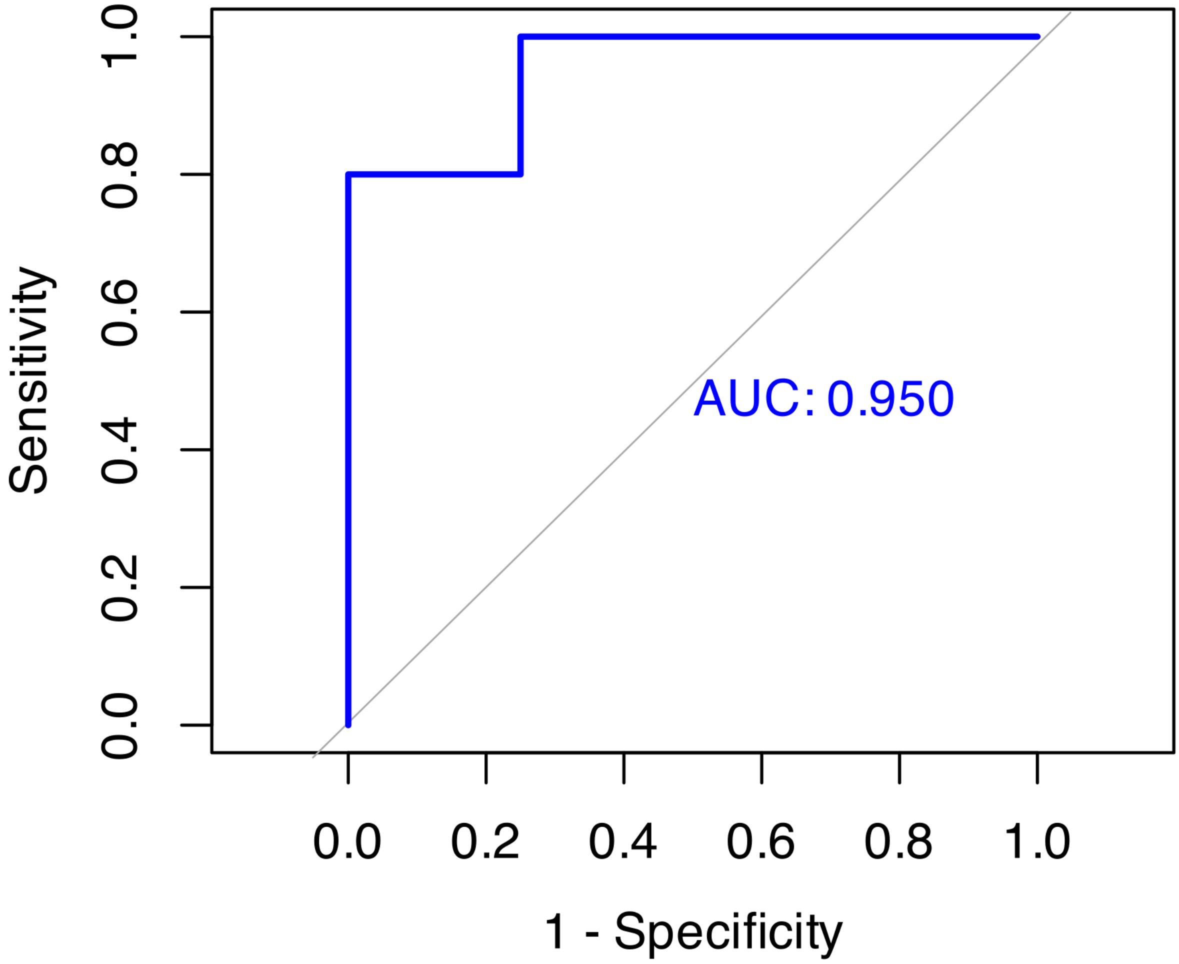

Supplementary Figure S2: ROC-AUC analysis of the combined classification score of the five candidate circRNAs (circ-CELSR1, circ-PLXNA1, circ-OBSL1, circ-DAB2IP, and circ-KANK1).
