# Supplementary material for: Postoperative Organ Dysfunction Risk Stratification Using Extracellular Vesicle-Derived circRNAs in Pediatric Congenital Heart Surgery
Source: Cells. 2024 Aug 25;13(17):1417. doi: 10.3390/cells13171417 (PMC11394075; doi:10.3390/cells13171417)
Supplement: Supplementary file 1 [file cells-13-01417-s001.zip › Table S1.pdf]

Supplementary Table S1: Age, gender, surgery type, and type of organ dysfunction in the clinical cohort

Patient ID	Age (m)	Gender	Surgery type	OD type
**OD1**	12	F	Double switch	Repiratory dysfunction, low cardiac output
**OD2**	4	F	PVS repair	Repiratory dysfunction
**OD3**	0.1	F	Norwood procedure	Repiratory dysfunction, low cardiac output
**OD4**	10	M	DORV repair	Repiratory dysfunction, low cardiac output
**NOD1**	0.1	F	Aortic arch & VSD repair	None
**NOD2**	12	F	VSD repair	None
**NOD3**	12	F	VSD repair	None
**NOD4**	8	M	Norwood procedure	None
**NOD5**	1	M	Aortic arch and VSD repair	None

Abbreviations: OD: Organ dysfunction; NOD: Non-Organ dysfunction; M: Male; F: Female m: month; PVS: Pulmonary Vein Stenosis; DORV: Double Outlet Right Ventricle; VSD: Ventricular septal defect.
