# Supplementary material for: Postoperative Organ Dysfunction Risk Stratification Using Extracellular Vesicle-Derived circRNAs in Pediatric Congenital Heart Surgery
Source: Cells. 2024 Aug 25;13(17):1417. doi: 10.3390/cells13171417 (PMC11394075; doi:10.3390/cells13171417)
Supplement: Supplementary file 1 [file cells-13-01417-s001.zip › Table S3.pdf]

Supplementary Table S3: Differential expression analysis of housekeeping EV miRNAs in OD versus NOD at post-surgery

	Base mean	log2(FC)	FDR
**miR-16-5p**	752.83	1.05	0.21
**miR-1228-3p**	2.14	0.98	NA
**let-7a-5p**	27449.34	0.94	0.34
**miR-23a-3p**	422.12	0.25	0.91
**miR-425-5p**	175.68	1.09	0.14

Abbreviations: miR: microRNA; FC: Fold Change; FDR: False Discovery Rate.
